# Targeted Phenolic Characterization and Antioxidant Bioactivity of Extracts from Edible *Acheta domesticus*

**DOI:** 10.3390/foods10102295

**Published:** 2021-09-28

**Authors:** Maria Catalina Nino, Lavanya Reddivari, Mario G. Ferruzzi, Andrea M. Liceaga

**Affiliations:** 1 Department of Food Science, Purdue University, 745 Agriculture Mall Drive West Lafayette, IN 47907, USA; mninober@purdue.edu (M.C.N.); lreddiva@purdue.edu (L.R.); 2 Department of Food, Bioprocessing and Nutrition Sciences, Plants for Human Health Institute, North Carolina State University, 600 Laureate Way, Kannapolis, NC 28081, USA; mferruzzi@uams.edu

**Keywords:** edible insects, phenolic compounds, antioxidant activity, bioactive compounds

## Abstract

With entomophagy gaining popularity in the Western hemisphere as a solution for future food insecurity, research on alternative protein sources, such as edible insects, has become relevant. Most of the research performed on insects has been on their nutritional qualities; however, little is known regarding bioactive compounds, such as polyphenols, that, if present in the insect, could provide additional benefits when the insect is consumed. In this study, methanolic extracts of *Acheta domesticus* from two farms and their corresponding feeds were obtained using a microwave-assisted extraction. Targeted phenolic characterization was accomplished through LC-MS/MS leading to the identification of 4-hydroxybenzoic acid, *p*-coumaric acid, ferulic acid, and syringic acid as major phenolic compounds in both *A. domesticus* extracts. Furthermore, the in vitro antioxidant activity was evaluated using 2,2-diphenyl-1-picrylhydrazyl radical cation (DPPH) and 2,2′-azino-bis (3-ethylbenzothiazoline-6-sulphonic acid) (ABTS) radical assays demonstrating the superior quenching activity of the *A. domesticus* extracts compared to the feeds. The discovery of phenolic compounds in *A. domesticus* implies the ability of this insect species to sequester and absorb dietary phenolics leading to possible added health benefits when consumed.

## 1. Introduction

Edible insects are becoming increasingly relevant as food insecurity and sustainability develop into a global concern. In 2013, the Food and Agricultural Organization (FAO), published the report ‘Edible insects: future prospects for food and feed security’ promoting insects as a key solution for food insecurity. This led to an increased interest in academia to generate applicable knowledge regarding entomophagy [1,2]. However, it is important to denote that entomophagy has limited acceptance due to it being an unfamiliar practice for almost all western cultures and even considered a cultural taboo, which has limited its incorporation in the current western diet [3]. In this context, and to provide further scientific-based information, research has been done on the sustainability, nutritional, and health benefits of edible insects [4].

For example, insects have been shown to contain high protein quantity and quality, even comparable to that of traditional livestock [5]. In addition, insect production has shown to be more sustainable as insects have a higher conversion efficiency, meaning that feed is more efficiently converted to body mass than conventional livestock [6,7,8,9]. For example, house cricket (*Acheta domesticus*), is twice as efficient as chicken, and 12-times more efficient than cattle [3]. Moreover, studies on mealworm (*Tenebrio molitor),* house cricket *(Acheta domesticus*) and migratory locust (*Locusta migratoria*) demonstrated that these three insects generated less greenhouse gas emissions, such as CO_2_ and CH_4,_ when compared to livestock and have reduced ammonia emissions, which is closely related with the nitrification and acidification of soils [6,8,10].

Among the different farmed edible insects, *A. domesticus* has gained momentum in the Western hemisphere due to the complete nutritional profile including a high protein content (64.4–70.7%, dry basis) [11]. In addition, these crickets can be a good source of vitamins and other micronutrients, such as potassium, calcium, iron, and magnesium [8,12]. Aside from the nutritional qualities, the biological activity of insect extracts has been of interest in recent years.

For example, del Hierro et al. [13] evaluated the in vitro antioxidant activity using the DPPH assay and the pancreatic lipase inhibitory capacity of *A. domesticus* and *T. molitor* extracts. Both insect extracts using ultrasound-assisted extraction (UAE) with ethanol:water had nearly 80% inhibition of the DPPH radical. In addition, the *T. molitor* extract was the most effective in inhibiting pancreatic lipase activity. Nevertheless, information regarding the bioactivity of *A. domesticus* extracts is scarce, and, to date, there is no published data regarding other bioactive compounds, such as polyphenols, present in *A. domesticus* or other edible insects consumed in the Western hemisphere.

Phenolic compounds are secondary plant metabolites characterized by the presence of one or more aromatic rings possessing at least one hydroxyl group attached to the aromatic structure [14]. Extensive research shows that phenolic compounds have several bioactivities, including antioxidant, anti-inflammatory, antimicrobial, and anticancer activities [15,16,17,18] among others, and diets rich in phenolics are associated with human health benefits. Given the insects’ herbivore feeding behavior and their complex interactions with plants, it would be expected that insects ingest plant phenolic compounds. In this matter, even though little research has been done, the available data show the ability of insects to sequester and metabolize plant phenolics from their diet [19].

Burghardt et al. [20] evaluated the flavonoid content of the common blue butterfly (*Polyommatus icarus*) identifying a selective absorption of quercetin and kaempferol from their host plants. Hirayama et al. [21] also identified two flavonol glycosides of quercetin and kaempferol from the cocoon of the white caterpillar (*Rondotia menciana*) that were fed exclusively with mulberry (*Morus alba*) leaves. These glycosides were not identified in the host plant implying that the insect was able to metabolize dietary flavonoids for further incorporation in the cocoon.

Despite the relevance of *A. domesticus* in entomophagy and the extensive research regarding the nutritional aspects of this insect, to our knowledge, no studies have been conducted on the characterization of phenolics or the role of the feed on the phenolic composition of farmed *A. domesticus*. This study aimed to first determine if the extracts from *A. domesticus* reared on two different commercial diets could contain phenolic compounds and whether these phenolics were related to their specific diets, and secondly, to determine if these extracts exhibit antioxidant activity.

Given the evidence of dietary phenolics in insects that has been reported in literature, as well as the evidence of in vitro antioxidant activity of insect extracts, our hypothesis is that *A. domesticus* extracts will have dietary phenolics and will exert antioxidant activity. Hence, the objective of the present study is to elucidate the phenolic composition of farmed *A. domesticus* consuming two different diets and evaluate their potential in vitro antioxidant activity.

## 2. Materials and Methods

### 2.1. Raw Materials and Chemicals

All materials and chemical reagents were purchased from Fisher Scientific (Waltham, MA, USA) and Sigma Aldrich (St. Louis, MO, USA), unless otherwise specified. The two *Acheta domesticus* samples (6-weeks old), organic *Acheta* and commercial *Acheta*, were obtained from two rearing farms (Aspire Food Group in Austin, TX and Ovipost, Inc. in Labelle, FL, USA), respectively.

Both cricket samples were shipped frozen and kept in a −20 °C freezer until needed. The organic feed was obtained from Aspire Food Group (Austin, TX, USA). The main ingredient composition of the organic feed consisted of organic corn, organic soybean, and organic alfalfa. The commercial feed used in the farm for the commercial *Acheta* was purchased from a commercial vendor (Mazuri^®^ Exotic Animal Nutrition, Richmond, IN, USA). The main ingredient composition of the commercial feed consisted of dehydrated alfalfa meal, wheat middlings, ground corn, ground soybean hulls, dehulled soybean meal, ground wheat, and dried beet pulp.

### 2.2. Preparation of Cricket and Feed Extracts

Feed and *A. domesticus* samples were freeze-dried for 72 h and then milled into a powder before processing. Microwave-assisted extraction (MAE) was done as described previously by Liu et al. [22] with a microwave accelerated reaction system (MDS, MARS-Xpress/230/60, CEM Corporation, Matthews, NC, USA). Briefly, 12 g of feed or cricket powder were suspended in 120 mL of petroleum ether while stirring for 30 min at room temperature. Then, the sample was microwaved at 900 W for 120 s at a controlled temperature of 40 °C.

The solvent was removed by filtration with a Whatman No. 1 filter, and the solid residue was collected for the next extraction step. The residue was then suspended in 120 mL of extraction solvent (methanol: d-water, 7:3 *v*/*v*), stirred for 30 min at room temperature and microwaved again at 900 W for 300 s at a controlled temperature of 50 °C. The solvent was collected and separated from the solid residue by filtration using a Whatman No.1 filter, then concentrated in a rotary evaporator at 55 °C, and finally freeze-dried for 48 h to obtain the extract. The freeze-dried extracts were stored at −85 °C in sealed containers until further use. The extraction was done in duplicate for each sample.

### 2.3. Total Phenolic Content of Extracts

The content of total phenolic compounds (TPC) of *A. domesticus* and feed extracts was determined in triplicate as described previously by Singleton and Rossi [23] with modifications by Cuadrado-Silva et al. [24]. Briefly, 35 µL of extract (10 mg/mL) or standard was mixed with 150 µL of a 1 N Folin reagent and was left to react for 5 min in dark conditions at room temperature in a 96-well plate. Then, 115 µL of a 7.5% (*w*/*v*) Na_2_CO_3_ solution was added, and the microplate was left in incubation at 40 °C for 30 min in darkness. After allowing the microplate to cool for 1 h, the absorbance at 765 nm was measured using a microplate photometer (Multiskan™ FC Microplate Photometer, Waltham, MA, USA). The results are expressed as g of gallic acid equivalents (GAE) per 100 g of extract using a standard curve of gallic acid (ranging from 40 to 240 µg/mL).

### 2.4. Phenolic Composition by UPLC/MS-MS

*A. domesticus* and feed extracts (10 mg/mL) were dissolved in 0.1% (*w*/*v*) formic acid in dd-water in preparation for solid-phase extraction (Oasis HLB extraction cartridges). The cartridges were activated using sequential passes of 1% formic acid in methanol and 1% formic acid in dd-water. The samples were loaded on the SPE cartridges and rinsed with 0.1% formic acid prior to polyphenol elution with 0.1% formic acid in methanol. Dried extracts were resolubilized in 0.5 mL of formic acid, dd-water, and methanol (0.1:49.9:50), filtered using a 0.45 μm PTFE filter, and analyzed by LC-MS/MS using a Waters Acquity I Class UPLC equipped with a XEVO TQD mass spectrometer (Waters, Milford, MA, USA).

The phenolics were resolved with an Acquity UPLC BEH C18 (2.1 × 50 mm) column at a flow rate of 0.5 mL min^−1^ using a gradient elution profile based on a binary phase of 0.1% formic acid in water (solvent A) and 0.1% in acetonitrile (solvent B). Separation was achieved at 40 °C using the following gradient: initially 100% A, 0–0.5 min 100–94% A, 0.5–2 min 94–91% A, 2–3 min 91–87% A, 3–4.5 min 87–65%A, 4.5–5.5 min 65–100% A, and 5.5–6 min 100% A. Phenolic compounds were detected under negative mode electrospray ionization (ESI-) with the following conditions: desolvation temperature 600 °C, desolvation gas flow 650 L h^−1^, capillary voltage 3 kV, cone voltage 32 V, and collision energy of 20 V. Multiple reaction monitoring (MRM) responses were used to quantify individual phenolic compounds.

### 2.5. Antioxidant Activity

Two in vitro assays were used to evaluate the *A. domesticus* and feed extract antioxidant potential. Freeze-dried extracts were reconstituted in distilled water, and four different concentrations (20, 10, 5, and 2.5 mg/mL for commercial *Acheta*, organic feed and commercial feed, and 5, 2.5, 1.25, and 0.625 mg/mL for organic *Acheta*) were tested. Trolox was used as a positive standard.

#### 2.5.1. DPPH (2,2-Diphenyl-1-Picrylhydrazyl Radical Cation) Radical-Scavenging Activity

DPPH was carried out as described by Reddivari et al. [25] with some modifications. First, a stock solution was prepared by dissolving 24 mg of DPPH in 100 mL ethanol. The working solution was prepared by diluting the stock solution until absorbance of 1.1 at 515 nm. Then, 15 µL of sample (*A. domesticus* or feed extracts) was mixed with 285 µL of DPPH working solution in a 96-well microplate. After incubation for 2 h in dark conditions, the absorbance at 515 nm was measured using distilled water as the control. Determinations were made in triplicate. The percentage of DPPH inhibition was determined with the following Equation (1):

% inhibition = ((A_control_ − A_sample_)/A_control_) × 100 (1)

IC_50_ values denote the concentration of sample required to scavenge 50% of the radical compound and were determined by interpolation from linear regression analysis.

#### 2.5.2. ABTS (2,2′-Azino-Bis 3-Ethylbenzothiazoline-6-Sulphonic Acid) Radical Scavenging Activity

The ABTS assay was developed following the method described by Ketnawa and Liceaga [26] with modifications. First, a 7 mM stock solution of ABTS radical was prepared in a 2.45 mM solution of potassium persulfate and left for incubation at room temperature for 16 h. After the incubation period, the working solution was prepared by diluting the stock solution with distilled water until absorbance of 0.7 at 734 nm. Then, 10 μL of sample (*A. domesticus* or feed extracts) was mixed with 294 µL of ABTS working solution in a 96-well microplate and was left in incubation in dark conditions at 30 °C for 10 min. Following the incubation period, the absorbance at 740 nm was measured using distilled water as a control. The IC_50_ values were determined using Equation (1) and by interpolation from linear regression analysis. Determinations were done in triplicate.

### 2.6. Protein Content

The total crude protein of the feed and *Acheta* samples was determined as N × 6.25 by the Kjeldahl method (AOAC methods 984.13 using a certified commercial laboratory (A&L Great Lakes Laboratories, Fort Wayne, IN, USA).

### 2.7. Statistical Analysis

All experiments and analyses were conducted in triplicate, unless otherwise indicated. The statistical analysis of the observed differences among means was performed using one-way analysis of variance (ANOVA), followed by Tukey’s pairwise comparison of means at a 5% significance level with the statistical software Minitab 18^®^ (State College, PA, USA).

## 3. Results and Discussion

### 3.1. Extraction Yields

The extract yields of A. domesticus and feed using microwave-assisted extraction (MAE) are given in Table 1. The extract yield of the organic *Acheta* and commercial *Acheta* extracts were 7.85% and 8.42%, respectively. In other insect species such as the rhinoceros beetle (*Allomyrina dichotoma*) the extraction yield with 80% methanol was 0.70% [27], which is much lower than the yields reported in the present study. In another study, the extraction yields of water and ethanol extracts of dark black chafer beetle (*Holotrichia parallela*) using MAE were reported as 25.03% and 10.22%, respectively.

In this case, the *H. parallela* water extract had a higher yield in comparison to our *A. domesticus* extracts; however, the ethanol extract yield appears to have a similar value to the yields reported in this study using the same extraction method [22]. The feed extracts had an extraction yield of 6.48% for the organic feed and 7.83% for the commercial feed, both similar to the *A. domesticus* yields. This similarity indicates that the extraction method can be used to obtain diverse compounds from plant as well as animal sources, since both types of samples had comparable yields. In addition, the microwave-assisted extraction method used in this study successfully extracted polyphenols allowing for the characterization of such compounds.

### 3.2. Total Phenolic Content of Extracts

A significant difference was observed (*p* < 0.05) for the total phenolic content of *A. domesticus* and feed extracts (Table 1). These values are in agreement with the values reported by del Hierro, Gutiérrez-Docio, Otero, Reglero and Martin [13] for *A. domesticus* ethanol extracts obtained my means of ultrasound-assisted extraction (UAE) and pressurized liquid extraction (PLE), where the total phenolic content was within a range of 0.3–5.0 g GAE/100 g of sample. Even though scarce information is available for edible insects, total phenolic content has been reported in recent studies.

Musundire et al. [28] reported a value of 3.6 g GAE/100 g for unprocessed edible stinkbugs (*Encosternum delegorguei*), where the authors also observed a decrease in total phenolics of the insect after applying traditional cooking techniques used in Zimbabwe. In a similar study, the total phenolic content of unprocessed edible beetle (*Eulepida mashona*) resulted in 0.08 g GAE/100 g of sample [29]. In another study, the total phenolic content of the edible ground cricket (*Henicus whellani*) was 0.77 g GAE/100 g [30], which is lower than the values reported in this study for *A. domesticus*.

Additionally, Liu, Sun, Yu, Zhang, Bi, Zhu, Qu, and Yang [22] reported 5 g GAE/100 g of sample for MAE extracts of *H. parallela*. This value is close to the phenolic content reported in this study for the *A. domesticus* extracts obtained using a similar extraction method (MAE). Similar to the *A. domesticus* extracts, the feed extracts were significantly different (*p* < 0.05) from each other, with the commercial feed having a higher value than the organic feed. Overall, the feed extracts had a lower total phenolic content in comparison to the *A. domesticus* extracts.

Given that the samples (insect and feed) are of different composition and the *A. domesticus* extracts represent a more complex matrix compared to the feeds, the higher total phenolic content in *A. domesticus* cannot be attributed only to the phenolic compounds present in the sample, but also to other components that can react with the Folin–Ciocalteu reagent. For example, the cricket extracts could contain unsaturated fatty acids, vitamins, and free amino acids that have shown reactivity with the Folin–Ciocalteu reagent in previous studies [31]. Despite the fact that the Folin–Ciocalteu assay is a well-known method to quantify phenolic compounds in diverse samples, the reaction mechanism is not specific for phenolics [32]. For example, other reducing compounds, such as ascorbic acid and select amino acids, could contribute to the total phenolic content, leading to an over-estimation of the phenolic compounds in the samples.

The crude protein was determined for each sample (Table 1). Even though no direct correlation can be made between the protein content and total phenolic content values, the relationship between these two variables is still observable. Both *A. domesticus* samples with higher protein content resulted in higher total phenolic content in comparison with both feeds that had less protein and lower total phenolic content, hence, resulting in the need to utilize more accurate and sensible methods, like the LC-MS/MS technique used in this study, to assess the phenolic content of the extracts [22,27].

### 3.3. Phenolic Composition of Extracts Using LC-MS

UPLC-MS/MS analysis allowed for the identification of 17 individual phenolic compounds in both feeds (organic and commercial); 13 were identified in the organic *Acheta* extract and 11 in the commercial *Acheta* extract (Table 2). Major compounds identified in both *Acheta* extracts correspond to 4-hydroxybenzoic acid, *p*-coumaric acid, ferulic acid, and syringic acid. A significant difference (*p* < 0.05) was observed in the concentration of *p*-coumaric acid and syringic acid, where the organic *Acheta* appears to have a higher concentration of both compounds. Even though the *Acheta domesticus* characterization shows very low concentrations of phenolics, these results can still be associated with their corresponding feeds.

Similar to the *Acheta* extracts, the same four phenolic acids constitute the major phenolic compounds identified in both feeds. No significant difference (*p* > 0.05) was observed between the feed extracts except for ferulic acid and chlorogenic acid, where the commercial feed had a higher concentration (Table 2). Given the presence of the same phenolic acids, including the same major compounds, were present in both sets of samples (*Acheta* and feed extracts), it is possible that farmed *A. domesticus* are able to absorb and sequester dietary phenolic acids from their feed. The confirmation of phenolic compounds in insects has been previously reported for a variety of species, mainly Lepidopterans (e.g., butterflies and moths), where the main assumption is that these compounds are directly obtained from the insects’ diet [20,33,34].

This hypothesis has been proven as some of the phenolics detected in the insect are also found in the host plants in which the insects are reared that constitute their primary feeding source [21,35,36]. Evidence of the absorption of dietary phenolic acids was reported previously in the literature. For example, Ferreres et al. [37] reported the presence of phenolic acids, ferulic, sinapic, and *p*-coumaric, in extracts of larvae of the large white butterfly (*Pieris brassicae*) reared on turnips (*Brassica rapa* var. *rapa* L). In a follow-up study, ferulic and sinapic acids were also found in this insect when reared on the Portuguese cabbage (*Brassica oleracea* var. *costata*) [38].

As reported, chlorogenic acid was detected in both feeds, being present at higher concentrations in the commercial feed. However, this compound was not found in the *Acheta* extracts. This could imply that *A. domesticus* is not able to absorb this compound or that it is metabolized and, hence, not detected. Evidence of excretion of phenolic compounds without being absorbed by the insect has been reported previously [33,38]. Further studies on the metabolism of plant phenolics by *A. domesticus* are needed to have more clarity on the fate of these compounds when ingested by the insect.

This study aimed to analyze if *A. domesticus* would be able to absorb dietary phenolics and, if so, how the composition of the feed influences the phenolic composition of *A. domesticus*. The composition of the commercial feed consisted of dehydrated alfalfa meal, wheat middlings, ground corn, ground soybean hulls, dehulled soybean meal, ground wheat, and dried beet pulp. In contrast, the organic feed consisted mainly of organic corn, organic soybean and organic alfalfa. Nevertheless, no difference in the phenolic composition of the feed was observed for the 17 individual phenolic compounds identified in this study (Table 2). Hence, no difference in the phenolic composition of the insects was expected.

The phenolic characterization showed a very low concentration of phenolic compounds for the insects as well as for the feed extracts. This might be attributed to the processing method of the feed. Both feeds require milling and drying processes that have positive as well as detrimental effects on the phenolic content of the raw plant material [39,40]. It is possible that the heating and grinding conditions utilized for the feed production, as well as the storage conditions and age of the final feed product, resulted in a decrease of the overall phenolic content on the feed, leading to low quantities of the phenolic compounds and, low quantities in the insects.

In contrast to the low quantities detected for the feed and *Acheta* extracts in this study (ng/10 mg), in other studies, the concentrations of the main phenolic compounds are in μg/g or mg/g [34,35]. This difference might be related to the insect’s diet as in previous studies the insects were reared in their host plants, fresh turnip and cabbage, which would have a higher concentration of phenolic compounds. Furthermore, the use of a processed diet instead of a fresh-plant diet could have an impact on the absorption of phenolics by the insect. Burghardt, Proksch, and Fiedler [20] observed that the absorption of phenolics was higher when *P. icarus* was fed natural host plants, compared to an experimental diet. Given that the farmed *A. domesticus* used for this study were fed with a processed diet instead of a fresh plant diet, the absorption of phenolics could have been affected.

As far as we know, this is the first time that the phenolic compounds in farmed *A. domesticus* fed two different processed and/or commercial diets have been reported. Because the insects used for this study were not starved before harvesting, it is unclear if the phenolic compounds were effectively absorbed or if the insect’s excrement composition is contributing to the overall phenolic content. Follow-up studies are necessary to confirm dietary phenolic absorption as well as the impact of sclerotization-derived phenolics in the overall phenolic composition of the insect.

Nonetheless, this approach allowed for the characterization in real conditions of the phenolic composition in farmed *A. domesticus* with the corresponding feed used by most edible insect farms in the USA. The results obtained in this study are of great interest since the discovery of these potential bioactive compounds in *A. domesticus* could potentially lead to added health benefits related to their consumption that were not previously contemplated. Further studies, especially regarding the bioavailability and bioaccessibility of these compounds could increase the interest on entomophagy, as this knowledge would represent an added value to insect consumption in addition to the nutritional characteristics (e.g., the protein content).

### 3.4. Antioxidant Activity

**Figure 1:**
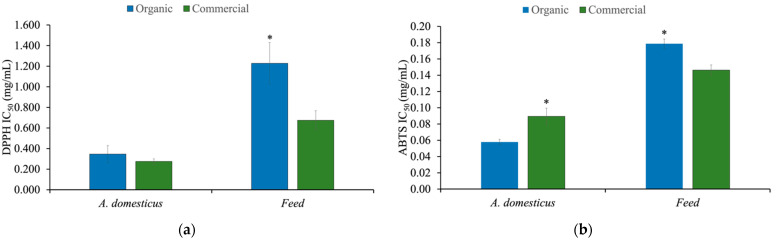
Antioxidant activity assays of extracts of *A. domesticus* (organic *Acheta* and commercial *Acheta*) and feed (organic feed and commercial feed): (**a**) DPPH radical scavenging activity (IC_50_ value, mg extract/mL); (**b**) ABTS radical scavenging activity (IC_50_ value, mg extract/mL). Asterisk (*) indicates significant difference (*p* < 0.05) between *A. domesticus* and feed extracts, respectively.

The potential antioxidant activity of the extracts was evaluated by their ability to inhibit DPPH and ABTS^+^ radicals. Although for both assays the antioxidant compound can exert its quenching activity by the hydrogen-atom transfer mechanism (HAT) or the single electron transfer mechanism (SET), SET appears to be the main mechanism occurring in the DPPH assay [41,42]. As the quenching ability of the antioxidant occurs through the same mechanisms in both tests, a comparison among them is acceptable. The antioxidant activity against the DPPH radical was superior for both *Acheta* extracts having IC_50_ values of 0.346 mg/mL and 0.275 mg/mL for the organic *Acheta* and commercial *Acheta*, respectively (Figure 1a).

The same result was obtained in the ABTS assay, where the *Acheta* extracts exhibited higher scavenging potential against this radical when compared to the feeds (Figure 1b), supporting the capacity of constituents of the extracts to act as primary antioxidants. The results obtained for the feed extracts showed a higher (*p* < 0.05) quenching ability by the commercial feed (DPPH IC_50_ = 0.674 and ABTS IC_50_ = 0.146) compared to the organic feed (DPPH IC_50_ = 1.228 and ABTS IC_50_ = 0.179).

The antioxidant potential of insect extracts has been previously evaluated using the DPPH radical assay showing promising antioxidant capacity. Suh, Kim, Lee, Park, and Kang [27] evaluated the antioxidant activity of Japanese rhinoceros beetle (*A. dichotoma)* extracts reporting moderate scavenging action depending on the extraction solvent with the lowest IC_50_ (0.119 mg/mL) for the methanolic extract.

Furthermore, aqueous MAE of *H. parallela* exhibited relevant antioxidant activity reporting an IC_50_ of 1.45 mg/mL [22]. The IC_50_ values obtained for *A. domesticus* in this study are similar to those reported for *A. dichotoma* when extracted with a similar solvent. However, they differ from the values reported for *H. parallela* using a similar extraction method. The similarity in the IC_50_ values could imply similar antioxidant activity with the *A. dichotoma* extracts, while the lower value compared to *H. parallela* could indicate the better antioxidant activity of the *Acheta* extracts.

The antioxidant activity of insect extracts has been previously attributed to the presence of phenolic compounds (mainly flavonoids) that have been long recognized to be potent antioxidants. Their antioxidant activity is due to the ability to neutralize free radicals, decompose peroxide species, and quench singlet and triplet oxygen [43], or as secondary antioxidants they can bind metal ions [42]. Nonetheless, it is also acknowledged that the presence of bioactive peptides, proteins and free amino acids may also contribute to the antioxidant activity of the extracts [27].

In addition, a correlation between the total phenolic content and DPPH scavenging activity has been recently reported for *T. molitor* and *A. domesticus* extracts, where those with the higher total phenolic content had higher DPPH inhibition [13]. As discussed previously, given that the Folin–Ciocalteu reagent is not selective to phenolics and uses tungsten ion to oxidize other compounds, like amino acid residues, it is likely that the observed bioactivity is a result of a group of bioactive molecules and not only phenolic compounds [27].

In this study, due to the low quantities of phenolic compounds detected in the *Acheta* extracts, the observed antioxidant bioactivity cannot be attributed only to the phenolic compounds that were characterized, but to a synergistic interaction between these compounds and proteins mainly, which are components found in the extracts (Table 1). Further studies on the characterization of other possible phytochemicals that could be present in the extracts, as well as peptides could lead to a better understanding of the bioactive potential of the insect extracts.

Although in vitro antioxidant activity serves as evidence of potential bioactivity of the extracts, this information is not sufficient to evaluate the real impact on human health, as evaluations of the bioaccessibility and bioavailability are needed. Future studies evaluating these parameters, including the use of simulated gastrointestinal digestion coupled with cell culture models (e.g., Caco-2-cells) could help to evaluate the bioaccessibility. Whereas, the use of in vivo models to evaluate the bioavailability (i.e., the fraction of the bioactive compounds that is absorbed and reaches the systemic circulation) could confirm the potential antioxidant benefits of insect consumption [44,45].

## 4. Conclusions

The data presented in this study confirms, for the first time, the presence of dietary phenolics in farmed *Acheta domesticus* (house cricket), suggesting that *A. domesticus* may be able to absorb or sequester dietary phenolics. The following phenolic acids, 4-hydroxybenzoic acid, *p*-coumaric acid, ferulic acid, and syringic acid were found to be the major phenolic compounds present in *A. domesticus* and feed extracts. In addition, microwave-assisted extracts of *A. domesticus* exerted in vitro antioxidant activity, higher than that exhibited by their plant-based feed extracts.

Based on the results observed in this study, phenolics as well as proteins may contribute to the antioxidant activity of the extracts. The results of this study suggest that farmed *A. domesticus* could potentially provide additional health benefits when consumed related to the composition of phenolics and other bioactive compounds. Nonetheless, due to the low quantities of these compounds present in the crickets and feed, further studies regarding bioavailability and bioaccessibility would be required to confirm their potential health benefits.

## Figures and Tables

**Table 1:** The total phenolic content (TPC) and crude protein of *A. domesticus* and feed extracts.

Extracts	Organic *Acheta*	Commercial *Acheta*	OrganicFeed	Commercial Feed
TPC (g GAE/100 g extract)	2.1 ± 0.05 ^a^	1.9 ± 0.04 ^b^	1.2 ± 0.08 ^x^	1.5 ± 0.08 ^y^
Crude protein (g/100 g)	45.13	43.88	9.88	13.94
Yield (%)	7.9 ± 0.27	8.4 ± 0.12	6.5 ± 0.02	7.8 ± 0.19

Total phenolic content (TPC) values (g GAE/100 g extract) are expressed as the mean ± standard deviation (*n* = 6). A comparison of TPC was made between the organic *Acheta* and commercial *Acheta*, and between organic feed and commercial feed, respectively. Different superscript letters indicate significant differences (*p* < 0.05) between samples. Protein content values are expressed as g/100 g of sample and were determined by a certified commercial laboratory. No SD values are shown for crude protein as only one analysis was made due to the small quantity of sample.

**Table 2:** Characterization of targeted phenolic compounds in *A. domesticus* and feed extracts by liquid chromatography coupled with electrospray-ionization triple quadrupole mass spectrometry (LC-ESI-QqQ-MS/MS).

Target Compound	RT (min)	Molecular Weight	MRM Transition	Phenolic Compounds (ng/10 mg)			
				Organic *Acheta*	Commercial *Acheta*	Organic Feed	Commercial Feed
**Phenolic Acids**							
Quinic acid	0.34	192	191 > 85	1.6 ± 0.9 ^a^	4.0 ± 2.2 ^a^	6.5 ± 0.9 ^x^	6.0 ± 1.5 ^x^
Gallic acid	0.64	170	169 > 125	1.4 ± 0.1 ^a^	0.5 ± 0.2 ^b^	6.3 ± 0.7 ^x^	9.2 ± 0.9 ^y^
4-hydroxybenzoic acid	1.28	138	137 > 93	29.4 ± 3.3 ^a^	20.6 ± 2.9 ^a^	79.9 ± 4.6 ^x^	70.3 ± 3.9 ^x^
Chlorogenic Acid	1.53	354	353 > 191	3.1 ± 0.7 ^a^	1.5 ± 0.7 ^a^	11.8 ± 1.3 ^x^	104.0 ± 13.2 ^y^
Caffeic acid	1.69	180	179 > 135	1.8 ± 0.3 ^a^	0.8 ± 0.1 ^b^	16.7 ± 1.6 ^x^	35.8 ± 4.5 ^y^
Syringic acid	1.98	198	197 > 167	13.8 ± 3.5 ^a^	4.7 ± 1.0 ^b^	127.3 ± 14.6 ^x^	132.8 ± 15.3 ^x^
p-coumaric acid	2.5	164	163 > 119	7.0 ± 1.3 ^a^	5.8 ± 0.7 ^b^	115.0 ± 9.1 ^x^	126.5 ± 15.7 ^x^
Ferulic acid	3.08	194	193 > 134	9.9 ± 1.2 ^a^	12.9 ± 1.6 ^a^	95.0 ± 5.4 ^x^	144.2 ± 9.1 ^y^
Sinapic acid	3.24	224	223 > 208	3.0 ± 0.2 ^a^	1.8 ± 0.2 ^b^	33.1 ± 3.7 ^x^	20.6 ± 2.3 ^y^
2-hydroxybenzoic acid	3.59	138	137 > 65	1.9 ± 0.2	n.d	20.0 ± 1.5 ^x^	12.8 ± 1.0 ^y^
**Flavonoids**							
Quercetin-3-glucoside	3.41	464	463 > 300	n.d	n.d	7.2 ± 0.8 ^x^	88.8 ± 4.7 ^y^
Quercetin-3-rutinoside	3.48	610	609 > 300	n.d	n.d	1.5 ± 0.1 ^x^	2.3 ± 0.2 ^y^
Kaempferol-3-glucoside	3.71	448	447 > 284	n.d	n.d	6.8 ± 0.8 ^x^	8.2 ± 0.5 ^x^
Daidzein	3.73	254	253 > 91	0.1 ± 0.0	n.d	7.4 ± 0.1 ^x^	5.6 ± 0.2 ^y^
Quercetin	4.28	302	301 > 151	1.3 ± 0.6	n.d	6.3 ± 0.6 ^x^	14.9 ± 0.9 ^y^
Naringenin	4.54	272	271 > 151	1.0 ± 0.1 ^a^	0.7 ± 0.1 ^b^	2.6 ± 0.2 ^x^	3.1 ± 0.3 ^x^
Apigenin	4.57	270	269 > 117	3.0 ± 0.2 ^a^	1.9 ± 0.0 ^b^	26.4 ± 2.4 ^x^	13.4 ± 0.4 ^y^

Values are expressed as the mean ± standard error (*n* = 6). Each phenolic compound was analyzed separately. A comparison was made between the organic *Acheta* and commercial *Acheta* and between the organic feed and commercial feed, respectively. Different superscript letters (a, b and x, y) indicate significant differences (*p*-value < 0.05) between sample groups (*Acheta* and feed, respectively); “n.d” implies the compound was not detected.

## Data Availability

The data presented in this study are available on request from the corresponding author. The data are not publicly available due to institutional privacy.
